# Imputation of low-coverage sequencing data from 150,119 UK Biobank genomes

**DOI:** 10.1038/s41588-023-01438-3

**Published:** 2023-06-29

**Authors:** Simone Rubinacci, Robin J. Hofmeister, Bárbara Sousa da Mota, Olivier Delaneau

**Affiliations:** 1 Department of Computational Biology, University of Lausanne, Lausanne, Switzerland; 2 Swiss Institute of Bioinformatics, Lausanne, Switzerland

**Keywords:** Software, DNA sequencing

## Abstract

The release of 150,119 UK Biobank sequences represents an unprecedented opportunity as a reference panel to impute low-coverage whole-genome sequencing data with high accuracy but current methods cannot cope with the size of the data. Here we introduce GLIMPSE2, a low-coverage whole-genome sequencing imputation method that scales sublinearly in both the number of samples and markers, achieving efficient whole-genome imputation from the UK Biobank reference panel while retaining high accuracy for ancient and modern genomes, particularly at rare variants and for very low-coverage samples.

## Abstract (web summary)

GLIMPSE2 is an improved method using sparse models for accurate, efficient and cost-effective genotype imputation in low-coverage whole-genome sequencing data.

## Main

Recent work and method advances^1–4^ highlight the advantages of low-coverage whole-genome sequencing (lcWGS), followed by genotype imputation from a large reference panel, as a cost-effective genotyping technology for statistical and population genetics. Large-scale whole-genome sequencing projects, such as the recent release of 150,119 samples from the UK Biobank^5^ (UKB), offer new opportunities to improve lcWGS imputation, potentially improving accuracy at rare variants (minor allele frequency (MAF) < 0.1%). However, current methods struggle to scale to the size of this new generation of reference panels resulting in prohibitive computational costs. To address this issue, we propose GLIMPSE v.2 (GLIMPSE2), a major improvement of GLIMPSE^1^, that scales to a reference panel containing millions of reference haplotypes, with high imputation accuracy at rare variants (MAF < 0.1%) and for very low-coverage samples (0.1× to 0.5×).

**Fig. 1:**
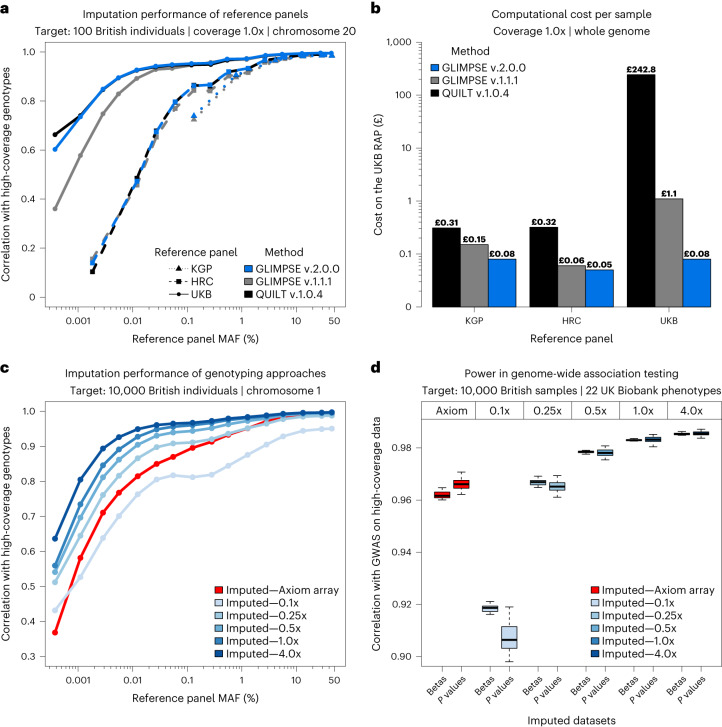
Accuracy, running time and power of low-coverage imputation using the UKB WGS data. **a**,**b**, Imputation performance of different imputation methods: QUILT v.1.0.4 (black), GLIMPSE1 (gray) and GLIMPSE2 (blue); across the 1,000 Genomes Project (KGP), HRC and UKB reference panels, for 100 UKB British samples at 1.0× coverage. **a**, Accuracy on chromosome 20 (Pearson *r*^2^, *y* axis), of imputation methods and reference panels: KGP (dotted line), HRC (dashed line) and UKB (full line). Accuracy is plotted against MAF of the appropriate reference panel (*x* axis, log scale). **b**, Cost per sample on the RAP for whole-genome imputation (*y* axis, log scale) across different reference panels (*x* axis). **c**,**d**, Performance of imputed data using the UKB reference panel across coverages (0.1–4.0×, different shades of blue, GLIMPSE2 imputation) and Axiom array data (red). **c**, Accuracy on chromosome 1 of 10,000 UKB British samples (Pearson *r*^2^, *y* axis) against MAF of the appropriate reference panel (*x* axis, log scale). **d**, Power in association testing of 10,000 UKB British samples compared to high-coverage data. Correlation of betas and *P* values (Pearson *r*^2^, *y* axis) of different imputed datasets (*x* axis) across 22 UKB phenotypes. Lower and upper limits of the box plots represent the first and third quartiles (Q1 and Q3); the median is marked at the center of the box. Lower and upper whiskers are defined as Q1 − 1.5 (Q3–Q1) and Q3 + 1.5 (Q3–Q1), respectively. Source data

To demonstrate the benefits of using sequenced biobanks for lcWGS imputation, we phased the recent release of the UKB WGS data^5,6^ using SHAPEIT5 (ref. ^7^) and created a UKB reference panel of 280,238 haplotypes and 582,534,516 markers (Supplementary Note 1). We used the UKB panel to impute lcWGS samples with GLIMPSE2 and other recently released imputation methods: GLIMPSE1 (ref. ^1^) and QUILT v1.0.4 (ref. ^2^). Compared to other reference panels, the UKB leads to considerable accuracy improvements for British samples across all tested depths of coverage. Furthermore, GLIMPSE2 outperforms GLIMPSE1, particularly at rare variants (MAF < 0.1%) and for very low-coverage (for 0.1× and 1.0× data at 0.01% MAF, GLIMPSE1 and GLIMPSE2 obtain an *r*^2^ of 0.561 and 0.892 compared to 0.725 and 0.927, respectively) and matches QUILT v.1.0.4 accuracy, designed to condition on the full set of reference haplotypes (for 0.1× and 1.0× data at 0.01% MAF, QUILT v.1.0.4 obtained an *r*^2^ of 0.728 and 0.925, respectively; Fig. 1a, Supplementary Note 2, Supplementary Figs. 1–3 and Supplementary Tables 2–4). We also find that the accuracy of GLIMPSE2 and QUILT v.1.0.4 methods is similar when imputing 42 non-European samples from 1,000 Genomes Project using the UKB reference panel (Supplementary Note 2, Supplementary Fig. 4 and Supplementary Table 5).

We further investigate the effect of the reference panel by imputing individuals of 129 human populations from the Simons Genome Diversity Project and we show that the UKB panel drastically improves imputation accuracy of European samples compared to the 1,000 Genomes Project reference panel, in particular of Northern Europe origin, for which the UKB reference panel obtains a reduction of non-reference discordance rate >67% (Supplementary Note 3, Extended Data Fig. 2 and Supplementary Fig. 8). Additionally, we imputed three ancient Europeans and a Yamnaya sample for which high-coverage data (>18×) are available and find similar improvements (Supplementary Note 4 and Supplementary Fig. 9), showing that some ancient populations, such as Viking, Western Hunter-Gatherer and Yamnaya could be well imputed from the UKB reference panel.

The imputation of a single lcWGS genome using the UKB reference panel is expensive or prohibitive using existing methods. On the UKB research analysis platform (RAP), the cost is £1.11 and £242.80 for GLIMPSE1 and QUILT v.1.0.4, respectively. In contrast, the same task performed with GLIMPSE2 only costs £0.08, due to major algorithmic improvements that drastically reduce the imputation time for rare variants (Fig. 1b, Supplementary Note 2 and Supplementary Figs. 5 and 6). We confirm this trend for up to 2 million reference haplotypes, using simulated data (Supplementary Note 2 and Supplementary Fig. 7). These improvements in imputation running time and memory requirements are crucial to keep lcWGS close to single nucleotide polymorphism (SNP) arrays in terms of computational costs^8,9^ (Supplementary Note 5) while maintaining the major advantage of providing better genotype calls. Indeed, we find that imputation of 0.5× data yields similar or more accurate results compared to the UKB Axiom array, with a notable difference at rare variants (for 0.5× coverage, accuracy improvement of *r*^2^ > 0.1 for variants with a MAF < 0.01%, Fig. 1c). Using simulated SNP arrays, we further confirm that 0.5× yields at least the same imputation accuracy as the densest SNP array model tested (Omni 2.5 array; Extended Data Fig. 3).

To assess the impact of these improvements on genome-wide association studies (GWAS), we imputed 10,000 UKB samples that we used to test 22 quantitative traits for association, comparing the respective abilities of lcWGS and SNP array data to recover the signals found with high-coverage sequencing data (Supplementary Note 6). We find that 0.5× leads to *P* values and effect size estimates as accurate as those obtained from Axiom array data (Fig. 1d and Supplementary Figs. 10–12) while delimiting regions of association with matching sensitivity and specificity (Supplementary Note 6 and Extended Data Fig. 4). We also look at rare loss-of-function, missense and synonymous variants^10^ and show that 1.0× outperforms the Axiom array for all categories of variants, an improvement that will be reflected in downstream burden-test analysis (Supplementary Note 7 and Extended Data Fig. 5). Altogether, this shows that lcWGS constitutes a powerful alternative to SNP array for downstream GWAS and rare-variant analysis.

In this work, we introduce several improvements to the GLIMPSE method that solve the computational problem of imputing lcWGS data from the 150,119 WGS samples in the UKB. We demonstrate that this reference panel leads to striking accuracy improvements across several sample ancestries, allele frequencies and depths of coverages. Our study further confirms the advantage of lcWGS over SNP arrays for GWAS, by showing that using imputed data with coverage as low as 0.5× are enough to outperform SNP array data, particularly at rare variants. Our work can be applied to other sequenced and diverse biobanks, such as Trans-Omics for Precision Medicine^11^, gnomAD^12^ or AllofUs^13^, thereby facilitating lcWGS imputation of non-European individuals. We believe that the difference between low-coverage and high-coverage WGS will become increasingly smaller as large reference panels will keep collecting more human haplotype diversity.

## Methods

This study relies on analyses of genetic data from the UKB cohort, which was collected with informed consent obtained from all participants. Data for this study were obtained under the UKB applications licence number 66995 and are available to registered researchers through the UKB data-access protocol. Additional data used in this study are all publicly available.

### GLIMPSE2

To perform imputation of low-coverage WGS data, GLIMPSE2 uses a Gibbs sampler algorithm that alternates between haploid imputation and phasing, using a modified version of the Li and Stephens hidden Markov model (HMM)^14^. The method necessitates a genotype likelihoods matrix for the target samples and a reference panel of haplotypes as input. The initialization step begins with the selection of a set of haplotypes from the reference panel via rare-variant calls derived from the low-coverage genotype likelihoods. Following that, two consecutive steps of haploid imputation are executed, one for each of the two target haplotypes. At the end of the initialization step, a diplotype is assigned to each target sample. GLIMPSE2 subsequently runs a series of burn-in and main Gibbs iterations to refine the genotype calls and phasing of each target sample. The algorithm determines haploid likelihoods for one of the two target haplotypes, based on the original genotype likelihoods and conditional on the current estimate of the other haplotype. To integrate over phasing uncertainty, the approach averages imputation posteriors across all main iterations.

Conversely from the GLIMPSE1 method, GLIMPSE2 approach is primarily focused on imputation only from the reference panel and it optimizes this task by incorporating new features. First, the reference panel is represented sparsely in memory, allowing for efficient storage of dense cohorts. The sparse representation of the reference panel facilitates the introduction of a new data structure to hasten haplotype matching and an efficient implementation of the HMM, which calculates posterior probabilities by leveraging the sparsity of the panel. Additional features of GLIMPSE2 include a genotype caller that integrates genotype likelihood computations directly into the GLIMPSE software and imputation of small insertions and deletions and low-quality variants separately from SNPs, by performing imputation into a haplotype scaffold obtained from high-quality SNPs.

The subsequent sections will provide a more comprehensive explanation of three of the previously referenced features, which are critical for the ability of the model to scale when applied to deeply sequenced reference panels. Further details regarding the method can be found in Supplementary Note 1.2.2.

### Sparse reference panel representation

GLIMPSE2 represents the reference panel as a sparse matrix, encoding haplotypes with one bit per allele if the variant is defined as common (MAF ≥ 0.001 by default) and storing the indices of the haplotypes that carry the minor allele, otherwise. This data representation allows for small memory usage but also for a fast identification of the haplotypes carrying a rare variant. Additionally, the transpose of the data structures gives efficient access to the rare variants of each haplotype. More details can be found in Supplementary Note 1.2.2.1.

We encoded the sparse reference panel representation in a binary file format to be efficiently stored on the disk. The file format translates directly into the memory data structures used by GLIMPSE2 and does not require any general-purpose compression algorithm. Together with the reference file format, we store the run-length encoded sparse positional Burrows–Wheeler transform (PBWT) data structure in the same file file, together with the recombination map. As a result, all the data related to the reference panel can be quickly loaded in memory, in much faster running times than standard file formats, such as VCF and BCF.

### Sparse positional Burrows–Wheeler transform matching

One of the key components of the GLIMPSE1 model is to reduce the state space using PBWT^15^, a data structure that allows efficient query searches in haplotype cohorts, linear in the number of samples and markers. Similarly, GLIMPSE2 extends the PBWT and proposes an algorithm designed for large sequencing cohorts, here called sparse PBWT.

**Extended Data Fig. 1:**
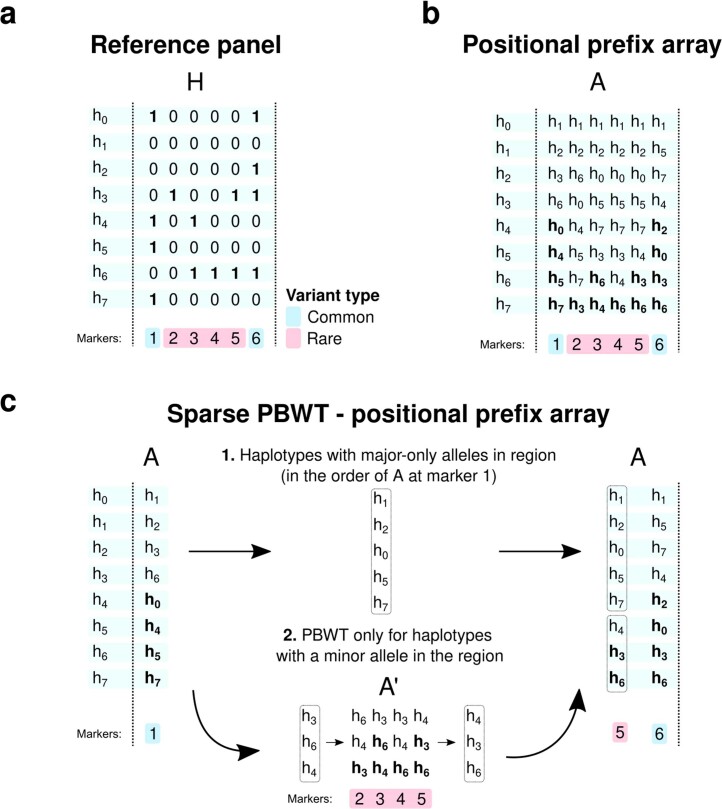
Sparse PBWT positional prefix array computation. **(a)** We consider a reference panel H with M = 6 markers and N = 8 haplotypes, h_0_,h_1_,…, h_7_. Here, marker 1 and marker 6 are common variants (light blue), and markers from 2 to 5 are rare variants (red). **(b)** Full prefix array A of the reference panel. **(c)** Sparse PBWT positional prefix array. At common variants (markers 1 and 6) the standard PBWT update is performed (light blue sites). At rare variants (red sites), no computation is required for the L = 5 haplotypes containing only the major allele in the region (h_0_,h_1_,h_2_,h_5_, h_7_) and they can be copied at the beginning of A_5_ in the same relative order as they appear in A_1_. For the haplotypes that contain the minor allele in the region (h_3_,h_4_,h_6_), we compute the positional prefix array A’at the rare variants in the interval. The last positional prefix array (A'_5_) can be directly copied into A_5_ from position N-L.

**Extended Data Fig. 2:**
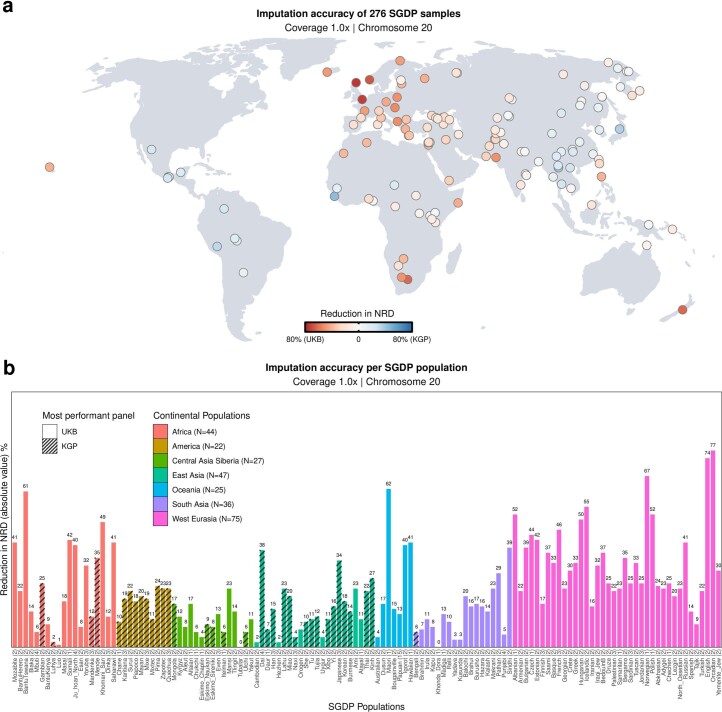
Imputation performance of SGDP samples using different reference panels. **(a-b)** Comparison between KGP and the UKB reference panels to impute 276 SGDP samples across 129 world-wide populations at 1.0x coverage on chromosome 20. (a) Per sample comparison. Each circle represents one sample of SGDP and is colored according to the reduction in NRD achieved when using the UKB reference panel (red) or KGP (blue). Location represents the geographical origin of the sample. (b) Population-level comparison. Samples belonging to the same population (x-axis) have been considered together (number shown in the x-axis label), showing the reduction of NRD between the two panels (y-axis). Populations have been colored and ordered according to the continent and country of origin. Striped bars represent populations where KGP performs better than UKB reference panels. Source data

**Extended Data Fig. 3:**
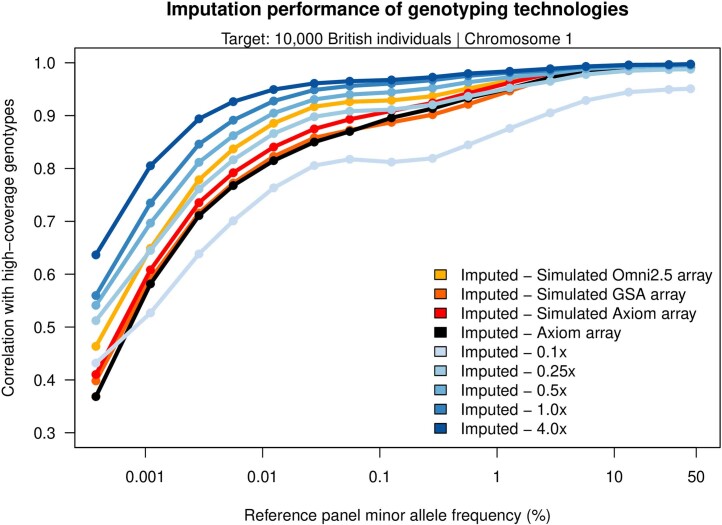
Imputation performance of simulated SNP arrays and low-coverage. Chromosome 1 imputation accuracy (Aggregate *r*^2^, y-axis) for 10,000 British samples of three simulated SNP arrays (Omni 2.5 Array, yellow; GSA array, orange; Axiom Array, red), and sequencing coverages (0.1–4.0x, shades of blue) using the UKB reference panel. The lifted-over (non-simulated) Axiom array data from the UK Biobank is shown in black. We imputed low-coverage data using GLIMPSE2 and SNP array data using BEAGLE v5.4. Source data

**Extended Data Fig. 4:**
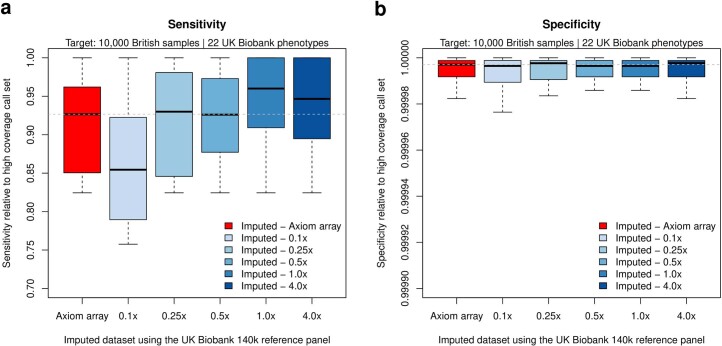
Sensitivity and specificity of genome-wide association using imputed call sets. **(a-b)** Sensitivity (**a**, y-axis) and specificity (**b**, y-axis) of GWAS by comparing with the validation GWAS across the 22 phenotypes examined. The x-axis shows the imputed call sets (0.1–4.0x, different shades of blue, GLIMPSE2 imputation; UKB Axiom array, red, imputed). Gray dotted lines represent the medians for GWAS using the Axiom array call set. The lower and upper limits of the box plots represent the lower and upper quartiles (Q1 and Q3); the median is marked at the centre of the box. Lower and upper whiskers are defined as Q1 − 1.5 (Q3–Q1) and Q3 + 1.5 (Q3–Q1), respectively. Source data

**Extended Data Fig. 5:**
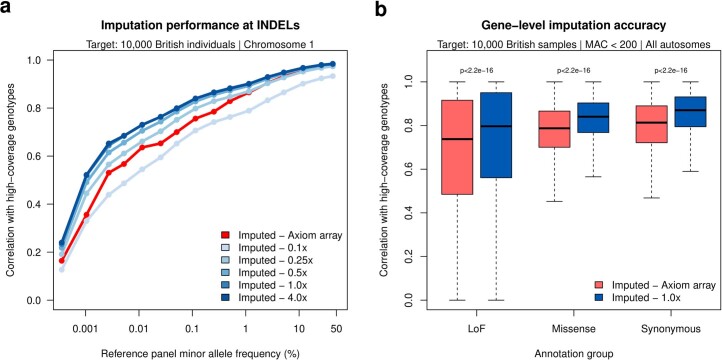
Performance at genomic annotations compared to high-coverage data. **(a-b)** Imputation performance of 10,000 British samples imputed using the UKB reference panel across coverages (0.1–4.0x, different shades of blue, GLIMPSE2 imputation) and the UKB Axiom array data (red). (a) Imputation accuracy at INDEL sites. (b) Gene-level imputation accuracy (Pearson *r*^2^, y-axis) at rare Genebass functionally annotated variants (LoF, loss of function; missense, synonymous variants; MAC < 200). Each data point represents a gene with at least one genetic variant across the 10,000 samples (defined *r*^2^ measure, N = 11185 Lof genes, N = 17003 missense genes, N = 17830 synonymous genes). P values between the imputed Axiom array and 1.0x data were computed with the two-sided Wilcoxon non-parametric rank sum test (LoF p-value = 1.9 × 10^−37^; Missense p-value < 5 × 10^−324^; Synonymous p-value < 5 × 10^−324^). The lower and upper limits of the box plots represent the first and third quartiles (Q1 and Q3); the median is marked at the centre of the box. Lower and upper whiskers are defined as Q1 − 1.5 (Q3–Q1) and Q3 + 1.5 (Q3–Q1), respectively. Source data

By using the sparse representation of the reference panel, rare variants are treated differently than common variants, allowing the computation of smaller PBWTs which speeds up the algorithm. This is based on the idea that between two adjacent common variants most of the haplotypes do not contain the minor allele in the region and therefore most of the haplotypes would form a single invariable block of major alleles that preserves their relative haplotype order. Therefore, a smaller PBWT is constructed only on haplotypes that have at least one minor allele between two adjacent common variants. The positional prefix array of the small PBWT at the end of the rare-variant interval is simply concatenated with the positional prefix array of other haplotypes that are not changing in the interval. A schematic illustration of the sparse PBWT is shown in Extended Data Fig. 1 and more details are provided in Supplementary Note 1.2.2.2.

Haplotype selection is performed by querying target samples in the sparse PBWT, looking at neighboring haplotypes at common variants (at 0.1 cM intervals by default). The selection is complemented with variant sharing at rare variants, as rare-variant sharing is likely to arise from a recent common ancestor.

### Sparse HMM computations

Imputation and phasing are performed using the forward–backward algorithm on the Li and Stephens HMM^14^, where reference haplotypes represent the states of the HMM. The computation of posterior probabilities is a computationally intensive task, linear in the number of haplotypes and markers.

The sparse matrix representation of the reference haplotypes in GLIMPSE2 implementation allows to remove the linear component at the marker level during the HMM calculations. GLIMPSE2 selects only $K$ (default $K=2,000$) haplotypes with the sparse PBWT selection to assemble a custom reference panel in which most of the rare variants present in the original reference panel are monomorphic. In the forward–backward algorithm these monomorphic variants do not contribute to the overall state probability. Therefore, in GLIMPSE2 the forward–backward probabilities are computed only at sites that are polymorphic in the custom reference panel, adjusting the transition probability to consider the physical distance between two consecutive polymorphic sites. Posterior probabilities of variants that are monomorphic in the custom reference panel can be quickly computed using the appropriate emission probability.

Our method takes advantage of low-level programming language (AVX2 intrinsics) to optimize the HMM forward–backward computations at the hardware level, working on blocks of eight floats. This allows the method to be efficient in the core part of the algorithm and therefore use twice the number of states and larger imputation windows compared to the previous version of GLIMPSE. More details are provided in Supplementary Note 1.2.2.3.

### Evaluation of imputation accuracy

We measured imputation performance as the squared Pearson correlation between high-coverage genomes and imputed dosages. We pooled all validation and imputed dosages belonging to the same frequency bin and computed a single squared Pearson correlation value per bin. Statististics summarizing the number of variants falling in each allele count bin are provided in Supplementary Tables 2–4. We used the GLIMPSE2_concordance tool to measure the squared Pearson correlation by streaming the imputed and validation data to maintain low memory requirements.

We also evaluated the non-reference discordance rate (NRD), defined as the rate between mismatches at the three possible genotypes, divided by the same mismatches plus heterozygous and homozygous alternative matches. We define the non-reference concordance rate as NRC = 1 − NRD. We provide more information about the benchmark and measurement of imputation accuracy in Supplementary Notes 1.3 and 1.3.1, respectively.

### Evaluation of association tests

We used chromosome 1 data for a subset of 10,000 unrelated UKB individuals of white British ancestry randomly sampled and a total of 99 phenotypes, selected as phenotypes with <10% of missing data in our call set across anthropomorphic traits and blood measurements. We performed association tests using plink2 (ref. ^16^) with default parameters and the first ten principal components plus sex and age as covariates to test phenotypes for associations with the seven call sets we generated: high-coverage WGS, five low-coverage WGS (0.1×, 0.25×, 0.5×, 1.0× and 4.0×) and the UKB Axiom array. We selected associations that are genome-wide significant (*P* < 5 × 10^−8^) and independent (being at least 500 kilobases apart). Out of the phenotypes analyzed, a total of 22 showed significant associations on chromosome 1 in the high-coverage dataset. These 22 phenotypes were chosen for comparison across the six imputed call sets.

To assess the accuracy of GWAS performed using imputed call sets, we compared association strength and effect sizes by computing the Pearson correlation between imputed and high-coverage GWAS experiments. We additionally assess the ability of GWAS experiments to distinguish significant from non-significant signals, considering the high-coverage GWAS to be the ground truth. For this, we computed the sensitivity, the proportion of genome-wide significant associations that can be retrieved, and the specificity, the proportion of genome-wide non-significant associations that can be retrieved using imputed call sets.

### Statistics and reproducibility

This study was based on the UKB SNP array and WGS datasets, Simons Genome Diversity Project, 1,000 Genomes Project and the Haplotype Reference Consortium (HRC). Variants and samples selected are based on quality controls and ancestry as described by the respective dataset. For certain analysis samples were extracted randomly from the UKB cohort, according to their ancestry. Statistical analyses, including Wilcoxon tests were performed with R v.4.0. All code to reproduce analyses is publicly available (Code availability section).

### Reporting summary

Further information on research design is available in the Nature Portfolio Reporting Summary linked to this article.

## Online content

Any methods, additional references, Nature Portfolio reporting summaries, source data, extended data, supplementary information, acknowledgements, peer review information; details of author contributions and competing interests; and statements of data and code availability are available at 10.1038/s41588-023-01438-3.

## Supplementary information

Supplementary Information Supplementary Notes 1–7, Figs. 1–12 and Tables 1–8.

Reporting Summary

## Data Availability

The 1,000 Genomes Project phase 3 dataset sequenced at high coverage by the New York Genome Center is available on the European Nucleotide Archive under accession no. PRJEB31736, the International Genome Sample Resource (IGSR) data portal and the University of Michigan school of public health ftp site (ftp://share.sph.umich.edu/1000g-high-coverage/freeze9/phased/). The publicly available subset of the HRC dataset is available from the European Genome-phenome Archive at the European Bioinformatics Institute under accession no. EGAS00001001710. The publicly available Simons Genome Diversity project is available on the IGSR data portal and Cancer Genomics Cloud, powered by Seven Bridges. The UKB WGS data and phenotypes can be accessed via RAP: https://ukbiobank.dnanexus.com/landing. The phased WGS reference panel can be accessed via RAP: https://ukbiobank.dnanexus.com/landing. Source data are provided with this paper.

## Source data

Source Data Fig. 1 Statistical source data.
Source Data Extended Data Fig. 2 Statistical source data.
Source Data Extended Data Fig. 3 Statistical source data.
Source Data Extended Data Fig. 4 Statistical source data.
Source Data Extended Data Fig. 5 Statistical source data.

## Code availability

GLIMPSE2 source code is available with MIT licence from https://github.com/odelaneau/GLIMPSE and https://odelaneau.github.io/GLIMPSE/. This includes code to the chunk, split_reference, phase, ligate and concordance. The documentation is available at https://odelaneau.github.io/GLIMPSE/. Code and source data to reproduce analysis and figures have been deposited in a Zenodo repository^17^.
